# Effect of Topical Anti-Glaucoma Medications on Late Pupillary Light Reflex, as Evaluated by Pupillometry

**DOI:** 10.3389/fneur.2015.00093

**Published:** 2015-04-29

**Authors:** Shakoor Ba-Ali, Birgit Sander, Adam Elias Brøndsted, Henrik Lund-Andersen

**Affiliations:** ^1^Research Laboratory, Department of Ophthalmology, Glostrup Hospital, Glostrup, Denmark; ^2^Faculty of Health and Medical Sciences, University of Copenhagen, Copenhagen, Denmark

**Keywords:** melanopsin, glaucoma, pupillometry, pupillary light reflex, latanoprost, timolol, dorzolamide, iris

## Abstract

**Purpose:**

The *late post-illumination pupillary response (PIPR*_10–30s_*)* to blue light is reduced in glaucoma, suggesting that pupillometry can be used in clinical glaucoma evaluation. Since animal studies have indicated that common anti-glaucomatous agents affect the iris muscle, we investigated the short-term effect of the anti-glaucoma drugs on the pupillary light reflex and in particular on the PIPR_10–30s_.

**Methods:**

In this randomized, double-masked, crossover trial, pupillometry was performed before and after topical administration of latanoprost, dorzolamide, and timolol in 20 healthy subjects. Stimulus was blue (463 nm) and red light (633 nm) of 2 log (lux). Main outcome was the PIPR_10–30s_ to blue light. Additionally, *pupil size*, *maximal contraction*, and the *early post-illumination pupillary response (PIPR*_0–10s_*)* to blue and red light were investigated. Pupil response variations between 8 a.m. and 2 p.m. were also assessed. *Intraocular pressure (IOP)* was measured before and 3.5 h after drug instillation.

**Results:**

We found no drug effect on the blue light PIPR_10–30s_ or any other blue light pupil parameters. During the control day, the only significant variation over time was observed for the red light PIPR_0–10s_ (*p* = 0.02). Pupillary size decreased slightly with timolol (0.1 mm, *p* = 0.03) and dorzolamide (0.2 mm, *p* < 0.001), but not with latanoprost. Timolol also reduced the maximal contraction amplitude significantly during red light (*p* = 0.02). Intraocular pressure was significantly reduced by all three drugs after 3.5 h (*p* < 0.01), while it remained unchanged during the control day (*p* = 0.3).

**Conclusion:**

Anti-glaucoma medications did not interfere with the blue light elicited PIPR. Dorzolamide reduced pupil size, while timolol reduced both pupil size and maximal contraction to red light, but the effect was minute and not of clinical importance.

## Introduction

Chromatic pupillometry is a relatively novel research tool for the evaluation of outer and inner retina function. The outer retina photoreceptors (rod and cones) exhibit fast temporal kinetics and cause a brisk pupillary constriction in response to light, while the inner retinal melanopsin containing intrinsic photosensitive retinal ganglion cells (ipRGCs) exhibit slower temporal kinetics and elicit a sustained pupillary constriction to light stimuli, persisting after light cessation (1). The melanopsin elicited sustained pupillary response after light offset is termed post-illumination pupillary response (PIPR) (2). Three studies, using chromatic pupillometry, have shown decreased pupillary response and in particular reduced PIPR in glaucoma patients, indicating functional impairment of the ipRGCs (3–5). Thus, pupillometry may be applied as a quick, non-invasive, and objective method to evaluate glaucoma progression. However, the pupillary light response is affected by various physiological and environmental factors – importantly, topical anti-glaucoma drugs may alter this response.

Animal studies have shown that latanoprost and other ocular hypotensive prostaglandin analogs such as travoprost, prostaglandin F2α, and bimatoprost have different affinities to FP-, E2-, and D2-prostaglandin receptor subtypes (6–10). These prostaglandin receptors cause contraction in cat and bovine iris sphincter muscle (6, 9, 10). Dinslage et al. found that latanoprost decreases constriction latency and pupil size in glaucomatous human eyes (11). Pindolol, a non-selective beta-blocker used as an anti-glaucoma agent, reduced resting pupil size and contraction amplitude of pupillary light reflex in healthy adults (12). Dorzolamide, a carbonic anhydrase inhibitor used in glaucoma, did not affect the pupillary light reflex (13, 14).

The main purpose of this study was to investigate the short-term effect of anti-glaucoma medications on the blue light elicited PIPR in healthy individuals.

## Materials and Methods

In this randomized, double-masked, crossover trial, the short-term effect of the three most frequently used anti-glaucoma eye drops (latanoprost 0.005%, timolol 0.5%, and dorzolamide 2%) on the pupillary light reflex were investigated by measuring the pupil response before and after administration of the eye drops.

### Subjects

Twenty-six healthy non-smokers aged 18–40 years with best-corrected visual acuity ≥1.0 were screened during April–July 2014. Exclusion criteria were ocular disease, systemic disease, refractive error >6 diopters, history of ocular surgery, use of medications, caffeine consumption during day of testing, pregnancy, psychiatric disease, lack of cooperation, smoking, and ocular abnormalities. Ophthalmologic examinations were performed to ensure normal conditions: Snellen visual acuity, Ishihara color vision, swinging flash light test, slit lamp examination, and intraocular pressure (IOP) measurement by Goldmann and Icare tonometer (Icare TA01i, Icare Finland Oy, Helsinki, Finland). In addition, visual field test (Octopus 900, 30°, HAAG-STREIT AG, Koeniz, Switzerland), non-mydratic fundus photography (Mark II Retinal Camera TRC-NW7SF, Topcon Corporation, Tokyo, Japan), and optical coherence tomography (OCT) scanning of the macula and optic nerve head (Spectralis OCT, Heidelberg Engineering GmbH, Heidelberg, Germany) were conducted. Arterial blood pressure, pulse, iris color, height, and weight were recorded. Six individuals did not meet the inclusion criteria. Young participants were included to create a homogenous study population and to counter any age-related bias, thus purifying the effect of drugs.

The trial was approved by Danish National Committee on Health Research Ethics (project ID: H-2-2014-029). Informed consent was obtained according to WMA Declaration of Helsinki’s ethical guidelines.

### Apparatus and experimental design

Pupillary response was recorded using a binocular multi-chromatic pupillometer (DP-2000 Human Laboratory Pupillometer, NeurOptics, Inc., CA, USA). This pupillometer consists of two separate integrated stimulation and recording units allowing for unilateral or bilateral eye stimulation while recording both the direct and the consensual pupillary response simultaneously. An adaptive eye cup prevents light scattering. The device is connected to a computer laptop with control software program.

The participants were assigned randomly to a sequence of latanoprost, timolol, and dorzolamide using a computer-generated randomization list including a fourth control test date without medication. Drugs were administered in a double masked fashion and washout period was minimum 1 week. In each experiment, pupil measurements were performed before (baseline), 30 and 180 min after drug application. Intraocular pressure was measured before and 3.5 h after medication. Measurements during the control visit were performed at equivalent hours. All experiments were conducted between 8 a.m. and 2 p,m. in April and September 2014 to avoid possible circadian and seasonal variations, respectively. The study eye for all participants was right eye.

**Figure 1:**
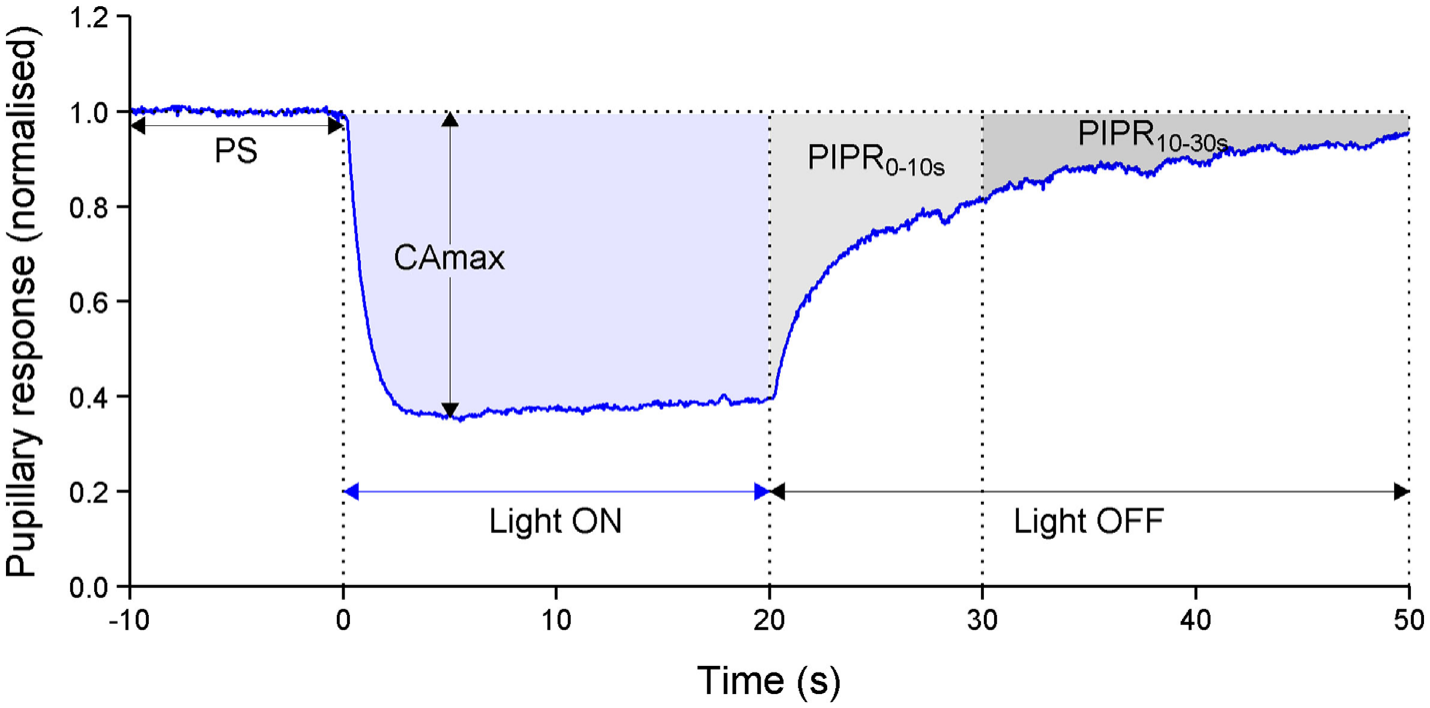
**An example of the pupillary light reflex to blue light (463 nm, 100 lux) and outcomes of this study**. The pupillary response is expressed as normalized. Time is in seconds (s). PS = mean of absolute pupillary diameter during the initial 10 s prior to light onset, CA_max_ = maximal contraction amplitude during 4–6 s of illumination time, PIPR_0–10s_ = mean of early post-illumination pupillary response 0–10 s after light offset and PIPR_10–30s_ = mean of late post-illumination pupillary response over a period of 10–30 s after light termination.

Prior to pupillometry, subjects were adapted to dark for 5 min. For each measurement, the pupil size was recorded for 10 s prior to light onset, 20 s during the light stimulation, and 60 s after the stimulus offset (Figure 1). First, participants were exposed to red light (633 nm, 2 log (lux), measured as 300 CD/m^2^) and 5 min later to blue light (463 nm, 2 log (lux), measured as 332 CD/m^2^). The binocular camera tracks and measures both pupils continuously with a frequency of 30 Hz. For both eyes, pupil diameters (millimeters), recording time (seconds), and luminance (lux) were recorded.

### Data processing and outcomes

Raw data were imported into the R-statistical package (version 3.1.0). An algorithm was used to detect and correct artifacts generated by eyelid blinks. If it was not possible to remove blinks by the algorithm, correction was performed manually.

The pupil diameter was normalized, i.e., expressed as the ratio relative to the baseline pupil size, and a total of three parameters were calculated to describe the pupillary response (15):

(1) The late post-illumination pupil response (PIPR_10–30s_), indicative of melanopsin photo pigment activation, was defined as the mean pupil constriction from 10 to 30 s after light termination (16).
(2) The early PIPR_0–10s_, a measure of mixed cone and melanopsin response, was defined as the mean pupil response from 0 to 10 seconds after light termination (16).
(3) Maximal contraction amplitude (CA_max_), a measure of cone response, was the maximum pupil constriction during 4 and 6 s of stimulation period (16).

Primary outcome was the PIPR_10–30s_ to blue light. Secondary outcomes, likewise for blue light, were the dark-adapted pupil size before light onset (PS), maximal contraction amplitude (CA_max_), and PIPR_0–10s_ (see Figure 1). For all the outcomes, the pupillary response to red light was also measured.

### Statistical analysis

SAS 9.3 and R-statistical package (3.1.0) were used to perform statistical analysis and graphics. Data were summarized as mean and SD for continuous variables, assuming normally distributed. The pupillary response was analyzed in relation to timing (before vs. after medication) and drug type with separate one-way analysis and the paired observations were accounted by a random statement (random coefficient model) of the mixed model procedure. The overall comparisons between drugs and control were performed with the procedure described above, followed by *post hoc* tests for pairwise comparison of the drugs and control; a Tukey statement was used to adjust for multiple comparisons.

## Results

### Subject

Final study population consisted of 20 subjects, 8 female and 12 male with mean age 25 ± 5.39 years. All subjects had normal visual field, color vision, and IOP (13 ± 2.24 mmHg). Mean body mass index was 24 ± 3.59 kg/m^2^ and iris color distribution was as the following: brown (55%), blue (35%), and green (10%).

### Outcome

In the following, for each outcome, *baseline examination* refers to pupillary measurements before medication at the start of each examination day, *effect of drug over time* is the comparison of pupillary response at baseline vs. after medication (30 and 180 min), and c*omparison between drugs and control* is the testing of the results for drugs against control measurements at 30 and 180 min (Tables 1–4).

**Table 1:** **Late post-illumination pupillary response (PIPR_10–30s_) to red and blue light measured at baseline and after (30 and 180 min) topical anti-glaucoma administration**.

	Mean ± SD			*p*-Value
	Baseline	30 min	180 min	Baseline vs. after
**Red light**				
Dorzolamide	0.052 ± 0.02^a^	0.053 ± 0.02	0.049 ± 0.02	*0.626*
Latanoprost	0.048 ± 0.01	0.047 ± 0.02	0.044 ± 0.01	*0.334*
Timolol	0.045 ± 0.01	0.047 ± 0.03	0.047 ± 0.01	*0.836*
Control	0.041 ± 0.02	0.044 ± 0.02	0.049 ± 0.01	*0.144*
*p-*Value	*0.021*	*0.327*	*0.664*	
**Blue light**				
Dorzolamide	0.153 ± 0.07	0.144 ± 0.07	0.145 ± 0.06	*0.662*
Latanoprost	0.156 ± 0.07	0.146 ± 0.06	0.139 ± 0.06	*0.347*
Timolol	0.156 ± 0.06	0.149 ± 0.06	0.139 ± 0.07	*0.290*
Control	0.158 ± 0.07	0.147 ± 0.06	0.147 ± 0.07	*0.516*
*p-*Value	*0.980*	*0.986*	*0.932*	

*Control refers to a visit, where no drugs were applied*.

*Results are presented as mean ± SD. *p*-Value < 0.05 indicates an overall significant change*.

*^a^Significant difference between the specific drug and control measurement*.

**Table 2:** **Early post-illumination pupillary response (PIPR_0–10s_) to red and blue light measured at baseline and after (30 and 180 min) topical anti-glaucoma administrations**.

	Mean ± SD			*p*-Value
	Baseline	30 min	180 min	Baseline vs. after
**Red light**				
Dorzolamide	0.175 ± 0.04	0.190 ± 0.04	0.184 ± 0.04	*0.069*
Latanoprost	0.178 ± 0.04	0.182 ± 0.04	0.173 ± 0.03	*0.340*
Timolol	0.172 ± 0.04	0.185 ± 0.05	0.181 ± 0.05	*0.182*
Control	0.180 ± 0.04	0.196 ± 0.04^a^	0.189 ± 0.03	*0.023*
*p-*Value	*0.507*	*0.299*	*0.157*	
**Blue light**				
Dorzolamide	0.307 ± 0.06	0.307 ± 0.06	0.307 ± 0.05	*0.999*
Latanoprost	0.305 ± 0.06	0.300 ± 0.06	0.294 ± 0.05	*0.534*
Timolol	0.312 ± 0.05	0.313 ± 0.06	0.306 ± 0.05	*0.692*
Control	0.323 ± 0.05	0.320 ± 0.06	0.319 ± 0.06	*0.878*
*p-*Value	*0.242*	*0.203*	*0.205*	

*Control refers to a visit, where no drugs were applied*.

*Results are presented as mean ± SD. *p*-Value <0.05 indicates the overall significant change*.

*^a^Significant difference in PIPR_0–10s_ measured at baseline and after (30 and/or 180 min) medication*.

**Table 3:** **Pupil size (PS) in millimeter at baseline and after (30 and 180 min) application with dorzolamide, latanoprost, and timolol**.

	Mean (mm) ± SD			*p*-Value
	Baseline	30 min	180 min	Baseline vs. after
**Prior to light**				
Dorzolamide	7.91 ± 0.72	7.82 ± 0.74	7.66 ± 0.71^b^	*0.0004*
Latanoprost	7.87 ± 0.65	7.87 ± 0.66	7.89 ± 0.65^a^	*0.808*
Timolol	7.92 ± 0.70	7.83 ± 0.76	7.79 ± 0.68^b^	*0.026*
Control	7.83 ± 0.70	7.78 ± 0.70	7.76 ± 0.71	*0.138*
*p-*Value	*0.104*	*0.247*	<*0.0001*	

*Control refers to a visit, where no drugs were applied*.

*Results are presented as mean ± SD. *p*-Value < 0.05 indicates the overall significant change*.

*^a^Significant difference between the specific drug and control measurement*.

*^b^Significant difference between measurement at baseline and after medication*.

**Table 4:** **Maximal contraction amplitude (CA_max_) to red and blue light measured before (baseline) and after (30 and 180 min) topical anti-glaucoma administration**.

	Mean ± SD			*p*-Value
	Baseline	30 min	180 min	Baseline vs. after
**Red light**				
Dorzolamide	0.523 ± 0.06	0.525 ± 0.06	0.527 ± 0.06	*0.412*
Latanoprost	0.534 ± 0.07	0.528 ± 0.06	0.520 ± 0.06	*0.193*
Timolol	0.522 ± 0.07	0.518 ± 0.06	0.501 ± 0.06^a^^,^^b^	*0.016*
Control	0.520 ± 0.08	0.530 ± 0.06	0.535 ± 0.05	*0.097*
*p-*Value	*0.354*	*0.413*	*0.001*	
**Blue light**				
Dorzolamide	0.622 ± 0.03	0.632 ± 0.03	0.632 ± 0.03	*0.082*
Latanoprost	0.628 ± 0.03	0.628 ± 0.03	0.626 ± 0.02	*0.949*
Timolol	0.631 ± 0.03	0.632 ± 0.02	0.625 ± 0.02^a^	*0.121*
Control	0.633 ± 0.03	0.636 ± 0.03	0.636 ± 0.02	*0.615*
*p-*Value	*0.236*	*0.295*	*0.046*	

*Control refers to a visit, where no drugs were applied*.

*Results are presented as mean ± SD. *p*-Value < 0.05 indicates the overall significant change*.

*^a^Significant difference between the specific drug and control measurement*.

*^b^Significant difference between measurement at baseline and after medication*.

### Late PIPR

#### Baseline examination

**Figure 2:**
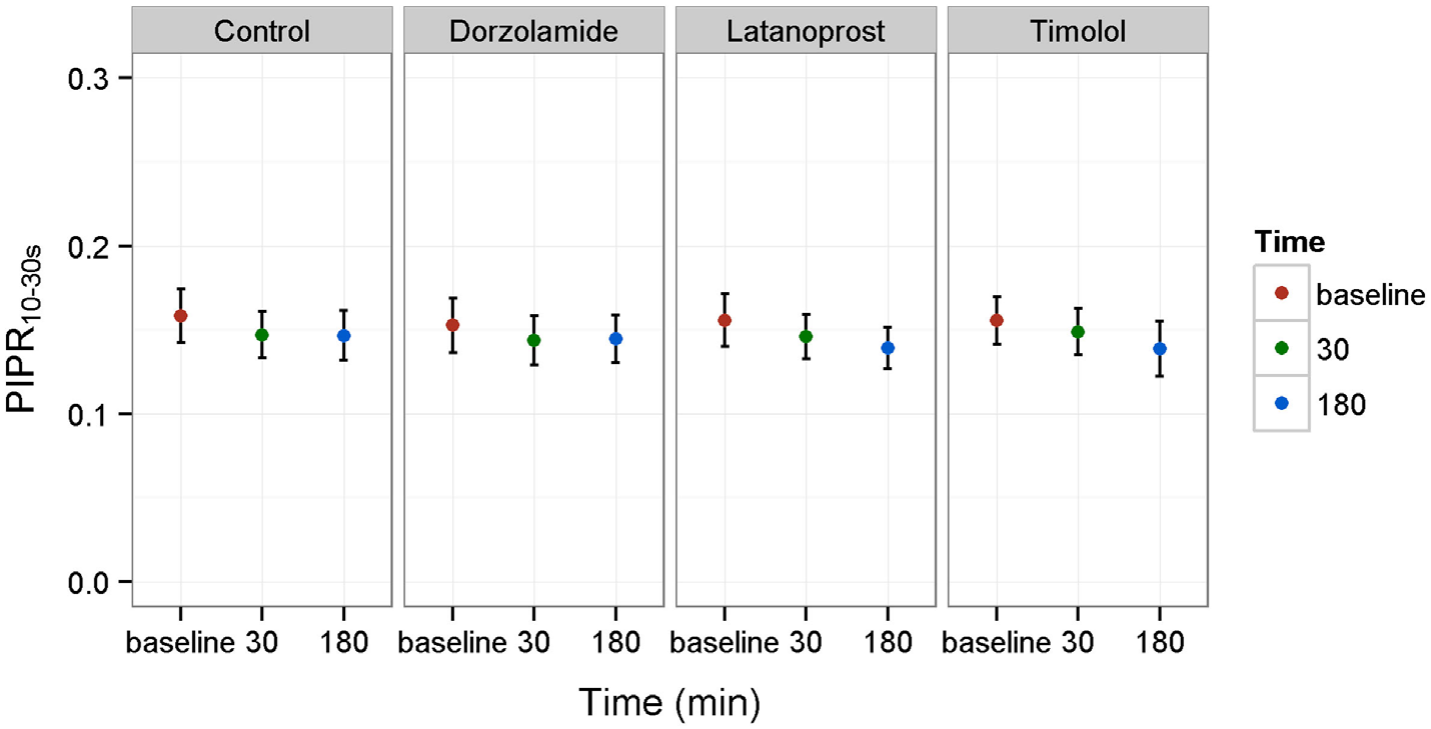
**Late post-illumination pupillary response (PIPR**_10–30s_**) to blue light and the effect of timolol, dorzolamide, and latanoprost**. Control refers to measurements without medication. PIPR_10–30s_ is presented as mean values ± SD. Time is given as baseline (base.) and after (30 and 180 min) drug application.

The late PIPR_10–30 s_ to blue light was similar during the days of examination (*p* = 0.980, Table 1; Figure 2). For red light, the response was significantly different (p = 0.021, Table 1). *Post hoc* analysis showed that the difference only applied to the day of dorzolamide application (*p* = 0.003).

#### Effect of Drug over Time

comparing baseline PIPR_10–30s_ to measurements after medication, none of the drugs changed PIPR_10–30s_ to blue or red light significantly (*p* > 0.1 for all data, Table 1).

#### Comparison Between Drugs and Control

No significant differences were found when comparing PIPR_10–30s_ measured during the days the participants received drugs and the control day (*p* > 0.1, Table 1).

### Early PIPR

#### Baseline Examination

The post-illumination pupillary constriction 0–10 s after light offset (PIPR_0–10s_) was similar in all examination days, both for red and blue light stimulation (red *p* = 0.507, blue *p* = 0.242, Table 2).

#### Effect of Drug over Time

The early PIPR_0–10s_ to both red and blue light was not significantly different between baseline and after topical instillation with timolol, dorzolamide, or latanoprost (*p* > 0.1 for all data, Table 2). We did see a significant increase in PIPR_0–10s_ during the control day after red light stimulation (*p* = 0.023), but not after blue light (*p* = 0.878). *Post hoc* analysis showed that the difference after red light was significant between baseline and 30 min, but not at 180 min (Table 2).

#### Comparison Between Drugs and Control

No significant differences were seen for either blue or red light comparing the PIPR_0–10s_ after drug applications against control measurements (Table 2).

### Pupil size

#### Baseline Examination

Pupil size (PS), measured prior to light onset, did not vary significantly at the start of each examination day, i.e., before drug administration (*p* = 0.104, Table 3).

#### Effect of Drug over Time

Dorzolamide and timolol reduced PS significantly (dorzolamide, *p* = 0.0004 and timolol, *p* = 0.026, Table 3). Latanoprost did not change the PS significantly (*p* = 0.808).

#### Comparison Between Drugs and Control

The overall comparison showed that PS was significantly different at 180 min (*p* < 0.0001), but not at 30 min. *Post hoc* analyses identified that pupil size was largest for latanoprost compared to control (0.12 mm, *p* = 0.026); dorzolamide and timolol had not significantly different effect compared to control values (Table 3).

To examine any carryover effect from red to blue light, additional analyses were performed to compare the PS prior to red light stimulation to that before blue light, as measured 5 min later. For the initial baseline examination, we found a statistically significant difference (*p* = 0.0232), however the difference was clinically negligible (0.05 mm, less than 1%). For the measurements at 30 and 180 min, the PS was unchanged between before red light illumination and prior to blue light (*p* > 0.05).

### Maximal contraction amplitude

#### Baseline Examination

CA_max_ to both red and blue light at the start of each examination days did not differ significantly (Table 4).

#### Effect of Drug over Time

CA_max_ to blue light did not change significantly before and after glaucoma medications, except for red light, where a significant decrease was found at 180 min after instillation with timolol (*p* = 0.016).

#### Comparison Between Drugs and Control

Both red and blue light were significantly different at 180 min (red light *p* = 0.001, blue light *p* = 0.046). *Post hoc* analysis showed that the decreased contraction amplitude was due to timolol (Table 4).

### Effect of covariates on PIPR and CA_max_

For all parameters mentioned above including *effect of drugs over time* and *comparisons between drugs and control*, the outcomes were analyzed after adjustment for baseline PS, age, gender, and iris color. For CA_max_ to blue light, the *p*-value did not reach statistical significance after adjustment for the baseline PS in the model thereby the *p*-value of 0.046 (Table 4) was reduced to 0.086. No other conclusions were changed. In addition, we did not find changes of the results due to the different randomization schemes of the drugs.

### Intraocular pressure

Intraocular pressure before and after drug application and for control measurements are summarized in Table 5. All three drugs reduced IOP significantly (*p* < 0.01) after 3.5 h, while the IOP during control day increased slightly, although not significantly (*p* = 0.3).

**Table 5:** **Intraocular pressure (IOP) measured before and 3.5 h after topical anti-glaucoma administration**.

	Mean ± SD		*p*-Value
	Before	After	
**Drugs**
Latanoprost	15 ± 2	11 ± 2	*<0.01*
Timolol	14 ± 2	10 ± 3	*<0.01*
Dorzolamide	15 ± 2	11 ± 3	*<0.01*
Control	13 ± 3	14 ± 2	*0.3*
*p*-Value	*0.02*	*<0.01*	

*Control refers to a visit, where no drugs were applied*.

## Discussion

Previous studies have shown that anti-glaucoma drugs affect the iris. The dilator and sphincter muscles of the iris regulate the pupillary size. The main objective of this study was to investigate whether the topical anti-glaucoma drugs can alter the PIPR to blue light in healthy individuals. This issue is important as pupillometry is gaining more ground in the evaluation of glaucoma. The approach was to compare the pupillary light response in healthy subjects before and after topical administration of timolol, dorzolamide, and latanoprost. As an additional control and to assess the variations of pupillary responses over a short time period (8 a.m. to 2 p.m.), we performed pupillometry during a control day, where no medication was applied (control).

Our results demonstrate that PIPR to blue light was unaffected by dorzolamide, latanoprost, or timolol (Tables 1 and 2) and no significant difference in hour-to-hour measurements through the control day was observed. The comparisons between drugs and control measurements during baseline examinations and after medications neither showed significant differences (Tables 1 and 2).

PIPR to red light was likewise unaffected by anti-glaucoma eye drops. There was a significant variation in the PIPR_10–30_*before* administration of dorzolamide in comparison to control measurement (Table 1). In addition, the PIPR_0–10s_ to red light increased through the control day without any medication (Table 2). These two statistically significant findings in relation to red light may have the following possible explanations. As reported earlier by Herbst et al., both the PIPR_0–10s_ and PIPR_10–30s_ to red light show low reproducibility (15). Previous studies have shown that PIPR to continuous light of both red and blue colors exhibits significant variations over 24 h (17, 18). Our measurements were performed from 8 a.m. to 2 p.m., and PIPR_0–10s_ to red light for both drugs and control increased during the three subsequent measurements throughout the daytime. Subjective sleepiness during the first measurement at the early morning could be an explanation for this increment, such that the test persons become more alert later on the day; Münch et al. showed a correlation between increased subject sleepiness and smaller PIPR (18), matching our findings, despite not recording the subjects” alertness during the day. Future experiments could begin with a trial stimulation to “awaken the subject” or could stimulate the pupil later in the day, e.g., commence at 10 a.m.

Since PIPR to blue light depends on the initial pupil size before light onset (19), we investigated the effect of anti-glaucoma eye drops on the PS. Dorzolamide reduced PS by 2.4% after 3 h (0.0004). Plummer et al. did not show any significant change in PS after application with dorzolamide in glaucomatous dogs (14). The inconsistency between our and Plummer’s findings could be explained by possible species differences in the effects of dorzolamide on the iris contractile muscle, in the same way as it was observed by Kaddour-Djebbar et al. for prostaglandin receptor agonist on the iris muscle (10).

Latanoprost did not change PS at 0.5 or 3 h (Table 3). Previous reports were contradictory, e.g., Dinslage et al. showed that latanoprost reduced PS in glaucomatous human eyes, while Marchini et al. did not report any significant change in glaucoma patients (11, 20).

Timolol reduced PS significantly 3 hours after medication (Table 3). This miotic effect was very small (1.64%), consistent with previous human studies, which also demonstrated a slightly non-significant decrease or no change of note in PS (21, 22).

The reducing effect of dorzolamide on the PS was also reported by Pfeiffer et al., although their results were statistically not significant (23).

The miotic effect of dorzolamide and timolol is an interesting finding since in case of substantial alteration in the PS, PIPR to blue light changed significantly as shown by Nissen et al. (19). For the results reported here, all conclusions remained unchanged after adjusting for the PS. Thus, dorzolamide and timolol have a slight, but clinical unimportant miotic effect.

The baseline examinations of the PS did not change significantly between examination days, indicating that no carryover effect was found from the drugs. We also evaluated if the pause of 5 min between red and blue light stimuli in our protocol was sufficient for the pupils to return to baseline; for the morning examination (i.e., baseline), a significant decrease in PS prior to blue light compared to PS before red light stimulation was observed. However, the difference was only 0.05 mm (<1%) and no differences were found for the examination at 30 and 180 min. Thus, 5 min is a sufficient time interval between light stimulations.

The PS did not change significantly during control measurements at 8 a.m. to 2 p.m. (Table 3), which is consistent with earlier reports investigating the PS variation over a short time period (17, 18).

Blue light CA_max_ during control day was consistent, revealing no variations from 8 a.m. to 2 p.m. in contrast to previous findings, Table 4 (17, 18). However, in our study, we did all the measurements between 8 a.m. and 2 p.m. to avoid the diurnal effect.

The difference observed between timolol and control in blue light CA_max_ at 180 min could be explained by modulations of CA_max_ over weeks since the time lag between control measurements and the examination day, we applied timolol, was minimum 1 week. In addition, the difference was not found when the model was adjusted for baseline PS.

Timolol reduced red light elicited CA_max_ by 3.85% after 180 min and moreover, there was a significant difference when comparing the drug against control (Table 4). Previous study reported similar effect of the non-selective beta-blocker pindolol on the contraction amplitude (12).

### Study limitations

One limitation of the present study was that we investigated the short-term effect of topical glaucoma medications. Latanoprost induces iris hyperpigmentation after continual use for approximately 7 months (24). However, while excessive doses of latanoprost may cause iritis, long-term trials do not indicate any adverse effects on the iris dynamics (25, 26). Dorzolamide has a very long terminal plasma elimination half-life (> 120 days), so for this drug, there could be a possible risk of drug effect several weeks following its instillation. To shed light on this issue, we investigated the carryover effect for the different drugs and found no significant change compared to baseline values. The long-term (1-year) adverse effects of timolol were studied very carefully by Sherwood et al. in a prospective trial – pupillary side effects were not reported (27).

Another limitation of the study is the role of preservatives in the three drugs. Since benzalkonium chloride (BAC) is added as preservative in all the three drugs, any possible effect of this preservative on the pupillary light reflex would not be identified in this short-term study. Benzalkonium chloride is shown to be a cytotoxic substance and cause apoptosis in conjunctival, corneal, and trabecular meshwork tissues (28–30). Although the penetration of BAC into the rabbit iris after topical instillation has been shown, its cytotoxic damage on the iris tissue has not been reported yet (31). To our knowledge, the adverse effect of the BAC on the iris has neither been investigated in pupillometry yet.

The age difference of our study population was 18–40 years, thus not representative of the common elderly glaucoma patients. Certain changes, possibly affecting the pupillary light response, occur with age: age-related miosis, photoreceptor degeneration, and decreased light transmission of especially blue light to the retina (32–34). However, currently three studies have investigated the effect of the age on the blue light elicited PIPR and neither of them showed any significant correlation between age and PIPR (2, 19, 35). In particular, Herbst et al. did not show correlation between age-related decrease in lens transmission and blue light elicited PIPR_10–30s_ (35). Moreover, in our younger study population, the effect of the drugs on the pupillary response was not biased by other possible age-related factors, which are not yet known. We do not expect any effect of glaucoma drugs on PIPR_10–30s_ in elderly individuals, such as glaucoma patients. However, our findings in a small number of young adults will need to be reproduced in a larger group of patients of older age, more typical of glaucoma patients, before the study can be applied directly to glaucoma patients.

## Conclusion

Our results demonstrate that PIPR (both PIPR_10–30s_ and PIPR_0–10s_) to blue light was *not* affected by anti-glaucoma medications. Red light elicited PIPR was not affected by topical glaucoma drugs either, however since the early PIPR_0–10s_ to red light increased significantly over a time period of 8 a.m. to 2 p.m. without medication, we suggest that this parameter is inconsistent and the results should be interpreted with caution. Dorzolamide decreased the dark-adapted PS, while timolol reduced both PS and CA_max_ for red light, but neither of these effects was of clinical importance.

## Conflict of Interest Statement

The authors declare that the research was conducted in the absence of any commercial or financial relationships that could be construed as a potential conflict of interest.

## References

[1] Kardon R Anderson SC Damarjian TG Grace EM Stone E Kawasaki A. Chromatic pupil responses: preferential activation of the melanopsin-mediated versus outer photoreceptor-mediated pupil light reflex. Ophthalmology (2009) 116(8):1564–73. 10.1016/j.ophtha.2009.02.007 19501408

[2] Kankipati L Girkin CA Gamlin PD. Post-illumination pupil response in subjects without ocular disease. Invest Ophthalmol Vis Sci (2010) 51(5):2764–9. 10.1167/iovs.09-4717 20007832 PMC2868495

[3] Kankipati L Girkin CA Gamlin PD. The post-illumination pupil response is reduced in glaucoma patients. Invest Ophthalmol Vis Sci (2011) 52(5):2287–92. 10.1167/iovs.10-6023 21212172 PMC3080733

[4] Feigl B Mattes D Thomas R Zele AJ. Intrinsically photosensitive (melanopsin) retinal ganglion cell function in glaucoma. Invest Ophthalmol Vis Sci (2011) 52(7):4362–7. 10.1167/iovs.10-7069 21498620

[5] Nissen CSB Milea D Kolko M Herbst K Hamard P Lund-Andersen H. Monochromatic pupillometry in unilateral glaucoma discloses no adaptive changes subserved by the ipRGCs. Front Neurol (2014) 5:15. 10.3389/fneur.2014.00015 24550887 PMC3914022

[6] Sharif NA Kaddour-Djebbar I Abdel-Latif AA. Cat iris sphincter smooth-muscle contraction: comparison of FP-class prostaglandin analog agonist activities. J Ocul Pharmacol Ther (2008) 24(2):152–63. 10.1089/jop.2007.0076 18355130

[7] Sharif NA Kelly CR Crider JY Williams GW Xu SX. Ocular hypotensive FP prostaglandin (PG) analogs: PG receptor subtype binding affinities and selectivities, and agonist potencies at FP and other PG receptors in cultured cells. J Ocul Pharmacol Ther (2003) 19(6):501–15. 10.1089/108076803322660422 14733708

[8] Ansari HR Kaddour-Djebbar I Abdel-Latif AA. Effects of prostaglandin F2alpha, latanoprost and carbachol on phosphoinositide turnover, MAP kinases, myosin light chain phosphorylation and contraction and functional existence and expression of FP receptors in bovine iris sphincter. Exp Eye Res (2004) 78(2):285–96. 10.1016/j.exer.2003.10.015 14729360

[9] Ansari HR Davis AM Kaddour-Djebbar I Abdel-Latif AA. Effects of prostaglandin F2alpha and latanoprost on phosphoinositide turnover, myosin light chain phosphorylation and contraction in cat iris sphincter. J Ocul Pharmacol Ther (2003) 19(3):217–31. 10.1089/108076803321908347 12828840

[10] Kaddour-Djebbar I Ansari HR Akhtar RA Abdel-Latif AA. Species differences in the effects of prostanoids on MAP kinase phosphorylation, myosin light chain phosphorylation and contraction in bovine and cat iris sphincter smooth muscle. Prostaglandins Leukot Essent Fatty Acids (2005) 72(1):49–57. 10.1016/j.plefa.2004.10.001 15589399

[11] Dinslage S Diestelhorst M Kuhner H Krieglstein GK. [The effect of latanoprost 0.005% on pupillary reaction of the human eye]. Ophthalmologe (2000) 97(6):396–401. 10.1007/s003470070087 10916381

[12] Smith SE Smith SA Reynolds F Whitmarsh VB. Ocular and cardiovascular effects of local and systemic pindolol. Br J Ophthalmol (1979) 63(1):63–6. 10.1136/bjo.63.1.63 367433 PMC1043389

[13] Pek H Wilhelm H. [Effect of dorzolamide on accommodation and pupillary reaction?]. Ophthalmologe (2000) 97(11):769–73. 10.1007/s003470070026 11130166

[14] Plummer CE MacKay EO Gelatt KN. Comparison of the effects of topical administration of a fixed combination of dorzolamide-timolol to monotherapy with timolol or dorzolamide on IOP, pupil size, and heart rate in glaucomatous dogs. Vet Ophthalmol (2006) 9(4):245–9. 10.1111/j.1463-5224.2006.00469.x 16771760

[15] Herbst K Sander B Milea D Lund-Andersen H Kawasaki A. Test-retest repeatability of the pupil light response to blue and red light stimuli in normal human eyes using a novel pupillometer. Front Neurol (2011) 2:10. 10.3389/fneur.2011.00010 21442043 PMC3057437

[16] Gamlin PD McDougal DH Pokorny J Smith VC Yau KW Dacey DM. Human and macaque pupil responses driven by melanopsin-containing retinal ganglion cells. Vision Res (2007) 47(7):946–54. 10.1016/j.visres.2006.12.015 17320141 PMC1945238

[17] Munch M Leon L Crippa SV Kawasaki A. Circadian and wake-dependent effects on the pupil light reflex in response to narrow-bandwidth light pulses. Invest Ophthalmol Vis Sci (2012) 53(8):4546–55. 10.1167/iovs.12-9494 22669721

[18] Zele AJ Feigl B Smith SS Markwell EL. The circadian response of intrinsically photosensitive retinal ganglion cells. PLoS One (2011) 6(3):e17860. 10.1371/journal.pone.0017860 21423755 PMC3056772

[19] Nissen C Sander B Lund-Andersen H. The effect of pupil size on stimulation of the melanopsin containing retinal ganglion cells, as evaluated by monochromatic pupillometry. Front Neurol (2011) 2:92. 10.3389/fneur.2011.00092 22319506 PMC3270334

[20] Marchini G Ghilotti G Bonadimani M Babighian S. Effects of 0.005% latanoprost on ocular anterior structures and ciliary body thickness. J Glaucoma (2003) 12(4):295–300. 10.1097/00061198-200308000-00002 12897573

[21] Gilmartin B Hogan RE Thompson SM. The effect of timolol maleate on tonic accommodation, tonic vergence, and pupil diameter. Invest Ophthalmol Vis Sci (1984) 25(6):763–70. 6724847

[22] Nordmann JP Mertz B Yannoulis NC Schwenninger C Kapik B Shams N. A double-masked randomized comparison of the efficacy and safety of unoprostone with timolol and betaxolol in patients with primary open-angle glaucoma including pseudoexfoliation glaucoma or ocular hypertension. 6 month data. Am J Ophthalmol (2002) 133(1):1–10. 10.1016/S0002-9394(01)01337-X 11755834

[23] Pfeiffer N Gerling J Lippa EA Brunner-Ferber FL Panebianco D Grehn F. Comparative tolerability of topical carbonic anhydrase inhibitor MK-927 and its S-enantiomer MK-417. Graefes Arch Clin Exp Ophthalmol (1991) 229(2):111–4. 10.1007/BF00170540 2044968

[24] Chou SY Chou CK Kuang TM Hsu WM. Incidence and severity of iris pigmentation on latanoprost-treated glaucoma eyes. Eye (2005) 19(7):784–7. 10.1038/sj.eye.6701663 15359238

[25] Alm A. Latanoprost in the treatment of glaucoma. Clin Ophthalmol (2014) 8:1967–85. 10.2147/OPTH.S59162 25328381 PMC4196887

[26] Linden C Alm A. The effect on intraocular pressure of latanoprost once or four times daily. Br J Ophthalmol (2001) 85(10):1163–6. 10.1136/bjo.85.10.1163 11567957 PMC1723731

[27] Sherwood MB Craven ER Chou C DuBiner HB Batoosingh AL Schiffman RM Twice-daily 0.2% brimonidine-0.5% timolol fixed-combination therapy vs monotherapy with timolol or brimonidine in patients with glaucoma or ocular hypertension: a 12-month randomized trial. Arch Ophthalmol (2006) 124(9):1230–8. 10.1001/archopht.124.9.1230 16966616

[28] De Saint Jean M Debbasch C Brignole F Rat P Warnet JM Baudouin C. Toxicity of preserved and unpreserved antiglaucoma topical drugs in an in vitro model of conjunctival cells. Curr Eye Res (2000) 20(2):85–94. 10.1076/0271-3683(200002)2021-DFT085 10617908

[29] Ayaki M Yaguchi S Iwasawa A Koide R. Cytotoxicity of ophthalmic solutions with and without preservatives to human corneal endothelial cells, epithelial cells and conjunctival epithelial cells. Clin Experiment Ophthalmol (2008) 36(6):553–9. 10.1111/j.1442-9071.2008.01803.x 18954319

[30] Baudouin C Denoyer A Desbenoit N Hamm G Grise A. In vitro and in vivo experimental studies on trabecular meshwork degeneration induced by benzalkonium chloride (an American Ophthalmological Society thesis). Trans Am Ophthalmol Soc (2012) 110:40–63. 23818734 PMC3671366

[31] Desbenoit N Schmitz-Afonso I Baudouin C Laprevote O Touboul D Brignole-Baudouin F Localisation and quantification of benzalkonium chloride in eye tissue by TOF-SIMS imaging and liquid chromatography mass spectrometry. Anal Bioanal Chem (2013) 405(12):4039–49. 10.1007/s00216-013-6811-7 23430186

[32] Daneault V Vandewalle G Hebert M Teikari P Mure LS Doyon J Does pupil constriction under blue and green monochromatic light exposure change with age? J Biol Rhythms (2012) 27(3):257–64. 10.1177/0748730412441172 22653894 PMC5380439

[33] Freund PR Watson J Gilmour GS Gaillard F Sauve Y. Differential changes in retina function with normal aging in humans. Doc Ophthalmol (2011) 122(3):177–90. 10.1007/s10633-011-9273-2 21562738

[34] Kessel L Lundeman JH Herbst K Andersen TV Larsen M. Age-related changes in the transmission properties of the human lens and their relevance to circadian entrainment. J Cataract Refract Surg (2010) 36(2):308–12. 10.1016/j.jcrs.2009.08.035 20152615

[35] Herbst K Sander B Lund-Andersen H Broendsted AE Kessel L Hansen MS Intrinsically photosensitive retinal ganglion cell function in relation to age: a pupillometric study in humans with special reference to the age-related optic properties of the lens. BMC Ophthalmol (2012) 12:4. 10.1186/1471-2415-12-4 22471313 PMC3411473

